# Impacts of agar gum and fucoidan on gel properties of surimi products without phosphate

**DOI:** 10.1002/fsn3.2973

**Published:** 2022-07-18

**Authors:** Mingjing Zheng, Jinling Hong, Pengjie Chuai, Yanhong Chen, Hui Ni, Qingbiao Li, Zedong Jiang

**Affiliations:** ^1^ College of Ocean Food and Biological Engineering Jimei University Xiamen China; ^2^ Collaborative Innovation Center of Seafood Deep Processing Dalian Polytechnic University Dalian Liaoning China; ^3^ Fujian Provincial Key Laboratory of Food Microbiology and Enzyme Engineering Xiamen Fujian China; ^4^ Research Center of Food Biotechnology of Xiamen City Xiamen Fujian China

**Keywords:** agar gum, fucoidan, gel properties, surimi

## Abstract

Phosphate is widely used in surimi products to improve the gel properties. However, excess addition of phosphate occurs, which can harm the consumer's health. This study aimed to evaluate the effects of agar gum and fucoidan on maintaining the gel properties of surimi products instead of phosphate. Interestingly, our results showed that 0.125% of agar gum and fucoidan to replace phosphate could enhance water‐holding capacity and maintain gel strength and textual properties of surimi products well. Especially at frozen storage for 1 year, 0.125% of agar gum reduced the expressible moisture content of surimi products by around 10% (*p* < .05). Sensory evaluation showed that 0.125% of agar gum and fucoidan instead of phosphate can improve tissue and fondness of surimi products in refrigerated storage for 24 h but not in frozen storage for 1 year. The addition of agar gum and fucoidan at a high concentration >0.50% increased the WHC, but significantly decreased gel strength and springiness of surimi products (*p* < .05). Particularly, 1.00% of agar gum and fucoidan reduced gel strength by around 20% (*p* < .05). It might be due to the destruction of the gel network structure of surimi protein following the excess addition of these polysaccharides. It can be concluded that 0.125% of agar gum and fucoidan can replace phosphate to develop high‐quality surimi products, and excessive addition of them have negative effects.

## Graphical abstract

Using agar gum and fucoidan to replace phosphate for producing high‐quality surimi products. Addition of 0.125% agar gum and fucoidan well maintained the gel properties of surimi products. Excessive agar gum and fucoidan destroyed gel structure of surimi proteins.

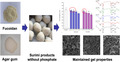

## 1 INTRODUCTION

Surimi is a myofibrillar protein concentrate made from fish meat. Its products (e.g., fish balls) are popular in the market due to their unique texture, savory tastes, and health benefits (Liu et al., 2019). Phosphate acts as a water‐retention and gel‐forming improvement agent and is widely used in aquatic products (Leloup et al., 1987; Park, 2000). Sodium pyrophosphate can improve water retention ability and gel strength of surimi (Julavittayanukul et al., 2006; Lee et al., 2018). However, excess addition of phosphate frequently occurs. High consumption of phosphates may be associated with several health problems including reduced calcium absorption and increased cardiovascular risk in humans (Teixeira et al., 2017). Thus, it is important to control the additive amounts of phosphates and identify suitable phosphate substitutes for surimi.

In addition to phosphates, polysaccharides like carrageenan and konjac glucomannan are generally adopted to improve the gel properties (e.g., gel strength, water‐holding capacity [WHC], and texture) of surimi (Iglesias‐Otero et al., 2010; Ji et al., 2017; Liu et al., 2013; Park, 2000; Zhang et al., 2018). Polysaccharides (e.g., agar gum, carrageenan, and fucoidan) are abundant in edible seaweeds, which possess excellent physicochemical properties (e.g., gelation, water retention, film formation, and emulsification) and good function (e.g., antioxidant, anti‐inflammatory, and bacteriostatic activities) (Salehi et al., 2019; Zhong et al., 2020). Seaweed polysaccharides have been used in surimi products. For example, as reported by Chen and Xue (2009), the addition of carrageenan and agar gum had beneficial effects on the gelation of horse mackerel surimi, while the addition of alginate only had a slight effect. Zhang et al. (2018) found that carrageenan could form an independent network to support and stabilize the reticulated framework of surimi proteins by filling interstitial spaces. Our previous studies revealed that an appropriate amount of fucoidan improved the gel strength and WHC of surimi products (Zheng et al., 2021). However, it is unknown whether such polysaccharides can maintain the WHC and gel properties of surimi products when used instead of phosphate.

Agar gum is a gelatinous and sulfated polysaccharide containing alternating β‐(1,3) and α‐(1,4)‐linked galactose residues and was the first phycocolloid employed in food and pharmaceutical applications (Atef et al., 2014; Xiao et al., 2019). Fucoidan is one of the water‐soluble sulfated polysaccharides with a fucose‐containing structure, which is commercially available as a nutritional supplement due to its good antiproliferative, antiangiogenic, and anticancer properties (Kwak, 2014). To determine whether seaweed polysaccharides can replace phosphate as a water‐holding agent in surimi products, 0.125%, 0.25%, 0.50%, and 1.00% (w/w) of agar gum and fucoidan were added to surimi products instead of phosphate, and then the gel properties and WHC of surimi products were evaluated. The Fourier transform infrared (FT‐IR) spectroscopy, sodium dodecyl sulfate‐polyacrylamide gel electrophoresis (SDS‐PAGE), and an environmental scanning electron microscope (ESEM) were performed to validate the proposed mechanism. Our findings will provide a theoretical basis for the production of high‐quality surimi products without phosphate and the high‐value practical use of seaweed polysaccharides.

## 2 MATERIALS AND METHODS

### 2.1 Materials

Freshwater surimi frozen at −20°C was provided by Fujian Anjing Foods Co., Ltd. (Xiamen, Fujian, China). Fucoidan (total sugar content, 68.22% ± 1.31%; fucose content, 41.10% ± 0.87%; sulfate content, 24.90% ± 0.15%; protein content, 6.50% ± 0.04%; and uronic acid, 3.80% ± 0.01%) was extracted from *Laminaria japonica* (obtained from Dongshan Island, Zhangzhou, Fujian, in summer) according to our previous method (Zheng et al., 2021). Agar gum (extracted from *Gracilaria*, purity 99%, food grade) was supplied by Shishi Globe Agar Industries Co., Ltd (Fujian, China). Phosphate, soybean oil, tapioca starch, and salt were commercially available.

### 2.2 Production of surimi products

The surimi products were produced using a previously reported method with minor modifications (Jia et al., 2019). The thawed surimi was cut into small cubes. The thawed surimi, soybean oil (10%, w/w surimi), tapioca starch (10%, w/w surimi), salt (2%, w/w surimi), phosphate compound (containing 0.01% sodium hexametaphosphate, 0.1% sodium tripolyphosphate, and 0.1% sodium pyrophosphate, w/w surimi), glutamine transaminase (0.25%, w/w surimi), and ice water (15% w/w surimi) were mixed using a ZB‐20 L chopper mixer (Chucheng Ruiheng Food Machinery Factory, Chucheng, China) at 1500 rpm for 2 min (i.e., the control surimi with added phosphate compound was labeled as C). Instead of a phosphate compound, different concentrations of agar gum and fucoidan (0.125%–1.00%, w/w raw materials) were added to surimi and other ingredients at 1500 rpm for 2 min. During the chopping process, the temperature was kept at <10°C by adding ice water to the double‐layer chopper. The surimi was shaped using a sausage filler, pre‐heated for 15 min at 45°C, and subsequently heated for 30 min at 90°C. After cooling with ice water, the surimi products of 3 cm pieces were prepared, and the casings removed. Some surimi products were refrigerated storage at 4°C for 24 h, and some surimi products were frozen storage at −18°C for 1 year.

### 2.3 Detection of expressible moisture content

The expressible moisture content (EMC) of the surimi products with refrigerated storage for 24 h and frozen storage for 1 year was detected using a previously reported procedure with some modifications (Barrera et al., 2002). Briefly, the surimi cubes, 2 mm in length, were put into a double‐layer filter paper, immediately centrifuged at 5000 × *g* for 10 min, and then the filter paper was removed. The calculation of EMC was based on Equation (1):

(1) $$\mathrm{EMC}\;\left(\%\right)=\left(W_{0}-W_{1}\right)/W_{0}\times 100\%$$

Where *W*_0_ is the weight of the initial surimi products, *g*; *W*_1_ is the weight of surimi products with expressible moisture removed after centrifugation, *g*.

### 2.4 Analysis of gel strength

A TA.XT2 Plus texture analyzer (Stable Micro System, Godalming Surrey, England) was adopted to determine the gel strength of the surimi products with refrigerated storage for 24 h and at frozen storage for 1 year following a previously reported procedure (Yan et al., 2021). The surimi cube of 3 cm in length was put on the platform. A P/5 s spherical probe was used to penetrate the surimi products into a depth of 2 cm at a rate of 1 mm/s. Both the pre‐test speed and post‐test speed were 4 mm/s, and the trigger force was 5 g. The gel strength of the surimi products was calculated using Equation (2):

(2) $$\text{Gelstrength}\left(g•\mathrm{cm}\right)=F\times D$$

Where *F* is the breaking force, *g*; *D* is the gel deformation, *cm*.

### 2.5 Determination of texture profile analysis (TPA)

The textural properties (e.g., hardness, chewiness, and springiness) of the surimi products with refrigerated storage for 24 h and frozen storage for 1 year were measured using the above TA.XT2 Plus texture analyzer based on a previous report (Li et al., 2021). The surimi products were analyzed under TPA model using a probe with 5‐mm‐diameter spherical plunger, and the penetration distance and rate were set at 0.5 mm/s and 20 mm, respectively. Finally, the hardness, chewiness, and springiness of the surimi products were obtained from the TPA curves.

### 2.6 Analysis of whiteness

The whiteness of the surimi products at refrigerated storage for 24 h and frozen storage for 1 year was detected using an ADCI‐WSI whiteness meter (Beijing Chentaike Instrument Co. Ltd., Beijing, China) based on a method of Benjakul et al. (2005). Whiteness was calculated using Equation (3):

(3) $$\text{Whiteness}=100‐{\left[{\left(100‐L^{*}\right)}^{2}+{a^{*}}^{2}+{b^{*}}^{2}\right]}^{1/2}$$

Where L* represents white (+) and black (−), a* represents redness (+) and greenness (−), and b* represents yellowness (+) and blueness (−).

### 2.7 Sensory evaluation analysis

Sensory evaluation was determined by six panelists using the method of Luo et al. (2022) with some modifications. The panelists have received professional sensory training. According to the sensory evaluation criteria in Table 1, the color, odor, tissue, springiness, taste, and fondness of the surimi products were evaluated.

**TABLE 1:** Sensory evaluation criteria

Project	Standard description	Score
Color	Gray‐green, with no gloss	0 ~ 4
	Yellow‐green, slightly shiny	5 ~ 6
	Light green, relatively shiny	7 ~ 8
	Gray, shiny	9 ~ 10
Odor	Strong fishy	0 ~ 4
	Obvious fishy	5 ~ 6
	Slightly fishy	7 ~ 8
	No fishy	9 ~ 10
Tissue	Cut surface with large pores, and uneven	0 ~ 4
	Cut surface with many small pores, and uneven	5 ~ 6
	Cut surface with tiny and uniform small pores	7 ~ 8
	Cut surface without small pores	9 ~ 10
Springiness	Cracks when pressed	0 ~ 4
	When pressed the depression does not crack, and the original shape does not return after press removal	5 ~ 6
	When pressed the depression does not crack, and can basically recover after press removal	7 ~ 8
	When pressed the depression does not crack and can rapidly recover after press removal	9 ~ 10
Taste	No fish taste and rough chew	0 ~ 4
	Less fish taste and slightly rough chew	5 ~ 6
	With fish taste, slightly delicate, and smooth	7 ~ 8
	With fish taste, delicate, and smooth	9 ~ 10
Fondness	Don't like	0 ~ 4
	General	5 ~ 6
	Prefer	7 ~ 8
	Like very much	9 ~ 10

### 2.8 FT‐IR analysis

The freeze‐dried surimi products with refrigerated storage at 4°C for 24 h were detected using an FT‐IR spectrometer (Nicolet iS50, Thermo Fisher Scientific, Waltham, MA) following a previous method (Zheng et al., 2021). The freeze‐dried surimi powder (10 mg) and potassium bromide (1000 mg) were mixed, evenly ground, and pressed into tablets, and scanned in the wavenumber range between 4000 and 400 cm^−1^.

### 2.9 SDS‐PAGE analysis

The SDS‐PAGE of surimi products with refrigerated storage at 4°C for 24 h was determined through electrophoresis (PowerPac Basic, Bio‐Rad Co., Hercules, CA) based on a previous report with some modifications (Benjakul et al., 2004). A mixture of surimi products (3 g) and 5% (w/v) SDS (27 ml) was prepared, homogenized, and heated for 1 h at 85°C to dissolve the protein. Then, the mixture was centrifuged to obtain supernatant at 8000 × *g* for 35 min at 4°C. Based on a previous report with slight modifications (Laemmli, 1970), the volume fractions of concentrated gel and separated gel were 5% and 10%, respectively. The loading volume of the above removed supernatant sample was 6 μl. The initial voltage was 100 V, which was changed to 80 V when the bromophenol blue moved to the junction of the concentrated gel and the separated gel. The polyacrylamide gel was stained using Coomassie Brilliant Blue R‐250, and decolorized using the acetic acid methanol solution.

### 2.10 Observation of surimi gel microstructure

The gel microstructure of the surimi samples with refrigerated storage at 4°C for 24 h was observed through the ESEM (Quanta 450, FEI Co., Hillsboro, OR) using a previously reported method with some modifications (Weng & Zheng, 2015). Briefly, the samples were cut into cubes (1 × 1× 1 cm), and then immersed in phosphate buffer (0.1 M, pH 7.2) with 2.5% (v/v) glutaraldehyde for 24 h at 4°C. Subsequently, the samples were washed with phosphate buffer (0.1 M, pH 7.2) three times, and then dehydrated with 30%, 40%, 50%, 70%, 80%, 90%, and 100% (v/v) ethanol solution in turn. Subsequently, all samples were then eluted in sequence using anhydrous ethanol and tert‐butanol mixtures with the ratios of 3:1, 1:1, and 1:3, and pure tert‐butanol. The prepared samples were frozen using liquid nitrogen, broken with a fracture knife, sublimated by gold plating, and then observed by the ESEM.

### 2.11 Statistical analysis

Six parallel experiments were performed for all analyses. The data are shown as mean ± standard deviation, which were examined through one‐way analysis of variance followed by Duncan's test using SPSS version 18.0. Statistical significance between different samples was set at *p* < .05. The presented figures are constructed using Origin 8.5.

## 3 RESULTS AND DISCUSSION

### 3.1 Impacts of agar gum and fucoidan on the WHC of surimi products

**FIGURE 1:**
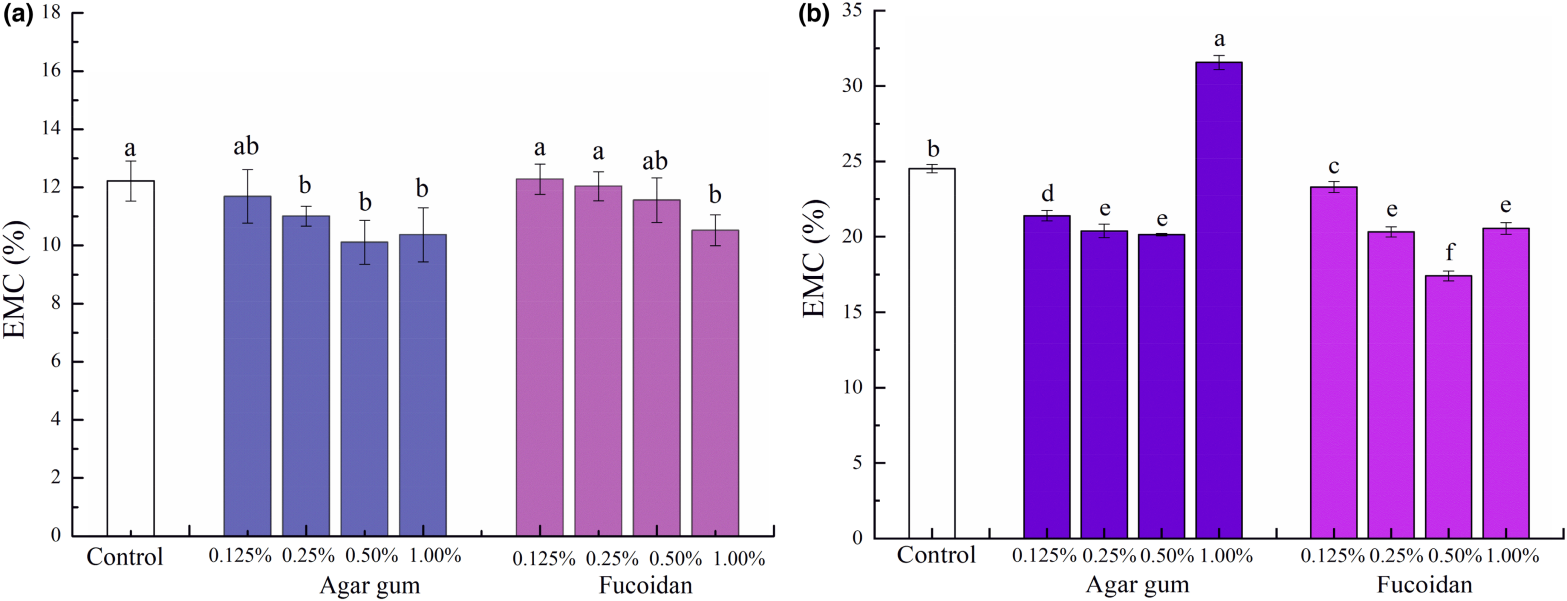
Impact of agar gum and fucoidan on the EMC of surimi products without phosphate: (a) at refrigerated storage for 24 h; (b) at frozen storage for 1 year.Control is the surimi products with phosphate. Different lowercase letters in the figure indicate statistical significance between different samples at *p* < .05.

The WHC of seafood products greatly influences the product yield and the functional and sensorial properties of the product (Alipour et al., 2018). The WHC of surimi gel can be determined by EMC, where the gel with better WHC generally displays lower EMC (Chaijan et al., 2006; Rawdkuen et al., 2004). The EMC of surimi products with added agar gum and fucoidan at different levels instead of phosphate is exhibited in Figure 1. After refrigerated storage for 24 h (Figure 1a), compared with surimi products with added phosphate, the EMC was slightly reduced when 0.125% of agar gum was added but the difference was not statistically significant (*p* ≥ .05). However, a significant decrease in EMC of surimi products was observed when the contents of added agar gum increased to 0.25%, 0.50%, or 1.00% (*p* < .05). The EMC of surimi products with 0.125%, 0.25%, or 0.50% of fucoidan added was similar to those with phosphate added (*p* ≥ .05), which greatly decreased until the level of fucoidan was 1.00% (*p* < .05). After frozen storage for 1 year (Figure 1b), the EMC of surimi products increased significantly as compared with the refrigerated one (*p* < .05). The increased EMC of surimi products in frozen storage is due to the migration of water molecules, formation, and growth of ice crystals during the freezing process, accelerating proteins aggregation, and disrupting the gel structure of surimi, thus to promote EMC (Otero et al., 2017). Compared to phosphate, agar gum and fucoidan were better in reducing the EMC of surimi products after frozen storage for 1 year (*p* < .05); except that 1.00% of agar gum significantly increased the EMC of surimi products (*p* < .05). Among phosphate compounds, appropriate sodium pyrophosphate can enhance water‐holding capacity and promote gel formation through enhancing protein cross‐linking, thus reduce EMC of surimi products (Julavittayanukul et al., 2006; Lee et al., 2018). The lower EMC of surimi products indicated that more water molecules may be bound or retained in the gel network as suggested by Chanarat and Benjakul (2013). The above results suggested that the addition of agar and fucoidan instead of phosphate can effectively maintain the WHC of surimi products except for 1.00% of agar gum after frozen storage for 1 year. Similar to a previous study reported by Petcharat and Benjakul (2018), the WHC of surimi products could be increased by the application of gellan, which decreased the EMC of surimi products, as gellan may absorb water molecules during the heating processing by charged domains and result in a reduction in the EMC. Thus, the increased WHC of surimi products may be in connection with the good water‐binding capacity of agar gum and fucoidan. Agar gum was observed to be better in increasing the WHC of surimi products than fucoidan, which may be dependent on the structural features of these two seaweed polysaccharides as suggested by a previous study (Zheng et al., 2019). However, stronger water‐binding properties of agar gum may intensify its water competition with proteins, resulting in increased EMC of surimi products with 1.00% of agar gum at long‐term frozen storage. Similarly, a previous study showed that the gel structure of surimi was disrupted due to the competitive hydration of the over‐added polysaccharides from green alga *Ulva intestinalis* (Alipour et al., 2018).

### 3.2 Impacts of agar gum and fucoidan on gel strength of surimi products

**FIGURE 2:**
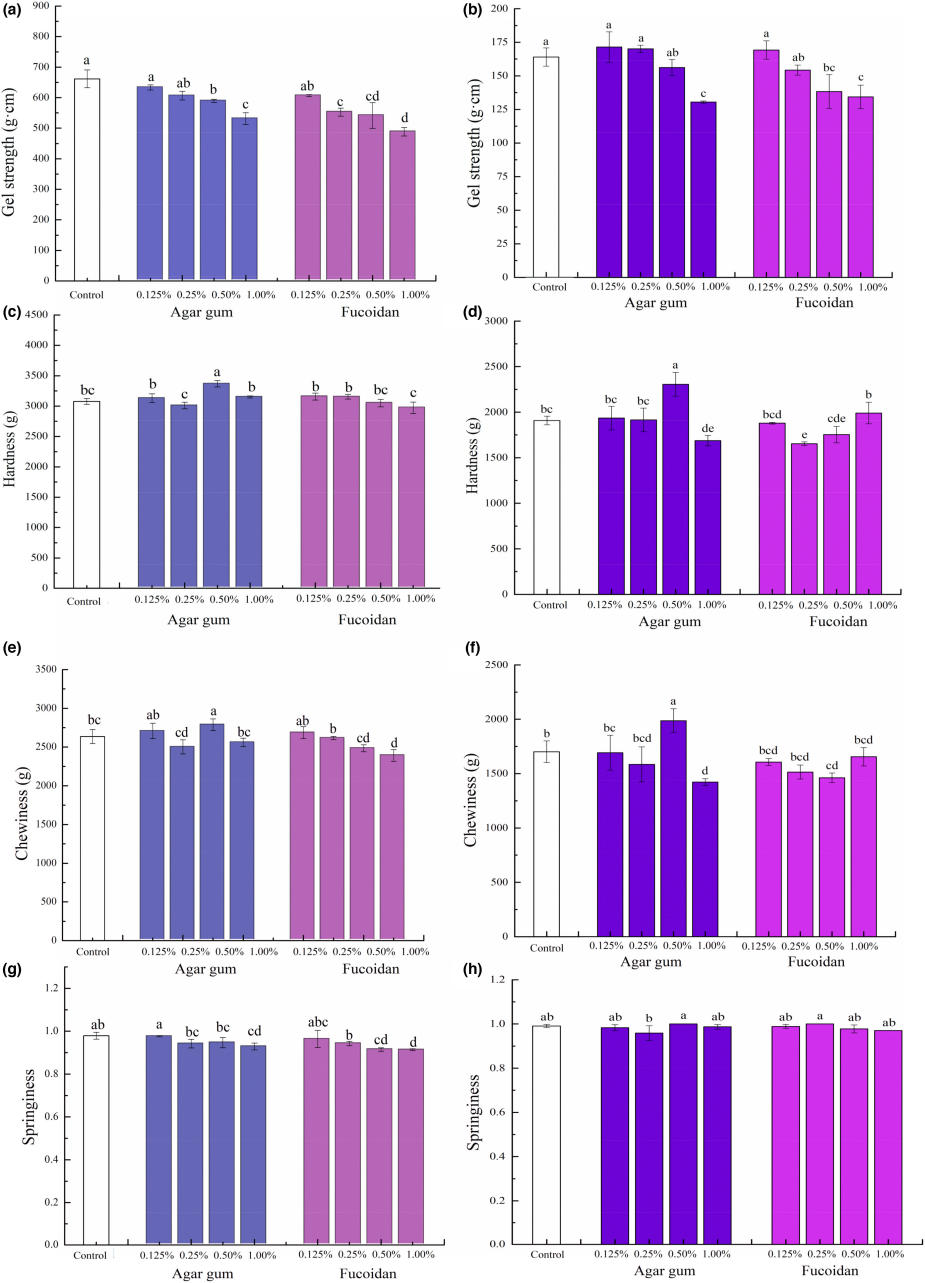
Impacts of agar gum and fucoidan on gel strength (a‐b) and textural properties (c–h) of surimi products without phosphate: (a, c, e, and g) at refrigerated storage for 24 h; (b, d, f, and h) at frozen storage for 1 year.Control is the surimi products with phosphate. Different lowercase letters in the figure indicate statistical significance between different samples at *p* < .05.

Gel formation is vital for the functional properties of food protein, which involves the formation of a network structure following protein denaturation and aggregation (Kinsella & Melachouris, 1976). Following the removal of phosphate, changes in the gel strength of surimi products with added agar gum and fucoidan are shown in Figure 2a,b. After refrigerated storage for 24 h (Figure 2a), the addition of 0.125% and 0.25% of agar gum instead of phosphate retained the gel strength of surimi products (*p* ≥ .05). But the gel strength of surimi products containing 0.50% and 1.00% of agar gum significantly decreased by about 10% and 20%, respectively (*p* < .05). Only 0.125% of fucoidan could maintain the gel strength of surimi products, which was equivalent to the function of phosphate. When the content of fucoidan was ≥ 0.25%, the gel strength of surimi products was significantly smaller than that of the control sample with phosphate added. In addition, its gel strength was also lower than that of surimi products with agar gum added at the same level (*p* < .05). These findings indicated that using no more than 0.25% agar gum and 0.125% fucoidan to replace phosphate could maintain the gel strength of surimi products at refrigerated storage for 24 h, and the effect was equivalent to that of phosphate. The maintaining effects of agar gum and fucoidan on the gel strength of surimi products to replace phosphate more obviously after frozen storage for 1 year (Figure 2b). Fucoidan at 0.25% could also replace phosphate to maintain the gel strength of surimi products (Figure 2b), while both 1.00% agar gum and 1.00% fucoidan decreased that by about 20%, suggesting that the appropriate dose of fucoidan can be selected in surimi products according to different storage conditions. However, the addition of agar gum and fucoidan at a higher level decreased the gel strength of surimi products. Similar to our findings, some polysaccharides such as xanthan gum and locust bean gum at a high level were found to negatively impact the mechanical properties of surimi products (Lee et al., 1992; Ramírez et al., 2002). Locust bean gum was used as a filler ingredient in surimi products, which influenced the gel formation of the continuous matrix, modified the character of the aqueous phase, and/or altered the textural properties of the products (Ramírez et al., 2002). It is assumed that the seaweed polysaccharides may act as filler ingredients to inhibit the gel formation, which was responsible for the decreased effect of agar gum and fucoidan at a high level on the gel strength of surimi products.

### 3.3 Impacts of agar gum and fucoidan on textural properties of surimi products

The impacts of agar gum and fucoidan on the textural properties of surimi products including hardness, chewiness, and springiness are illustrated in Figure 2c–h. The effects of agar gum and fucoidan on the textural properties of surimi products without phosphate after frozen storage for 1 year were similar to those refrigerated in storage for 24 h. As shown in Figure 2c,d, the hardness of surimi products with 0.125%, 0.25%, and 1.00% of agar gum added instead of phosphate was close to that of control surimi products with phosphate added (*p* ≥ .05). However, adding 0.50% of agar gum greatly increased the hardness of surimi products, which was around 10% when compared with that of control with phosphate added (*p* < .05). The effects of 0.125% of fucoidan on the hardness of surimi products were close to the impact of phosphate, but 0.50% and 1.00% of fucoidan led to a reduction in the hardness of surimi products (*p* < .05). The chewiness of surimi products was influenced by agar gum and fucoidan at different levels (Figure 2e,f) and was consistent with the effect observed on hardness. Our results showed that using 0.125% and 0.25% of agar gum and fucoidan instead of phosphate effectively maintained the chewiness of surimi products (*p* ≥ .05). With regard to the springiness of surimi products (Figure 2g,h), the addition of 0.125% of agar gum and fucoidan to replace phosphate well maintained the springiness of surimi products at refrigerated storage for 24 h, and such effects were more obvious after frozen storage for 1 year. Also, adding only 0.25% and 0.50% of agar gum, as well as fucoidan, slightly reduced the springiness of surimi products, but the difference was not statistically significant (*p* ≥ .05), while the springiness of surimi products significantly decreased when 1.00% of agar gum and fucoidan was added (*p* < .05). The results are well consistent with those of a previously reported study that investigated the application of isolated sulfated polysaccharides extracted from the green alga *Ulva intestinalis* (UIP) in surimi products. It was reported that the hardness, chewiness, and springiness were similar between control and surimi products containing UIP at a level of 0.25% (*p* > .05), and a significant lower trend was found in hardness and chewiness when UIP concentrations were higher than 0.25% (*p* < .05) (Alipour et al., 2018). The reason for reduced gel hardness and chewiness at high doses > 0.25 g/100 g of sulfated polysaccharides was because of a repulsive interaction between polysaccharides and surimi proteins and higher affinity of polysaccharides to bind water molecules with respect to proteins (Alipour et al., 2018).

### 3.4 Impacts of agar gum and fucoidan on whiteness of surimi products

**FIGURE 3:**
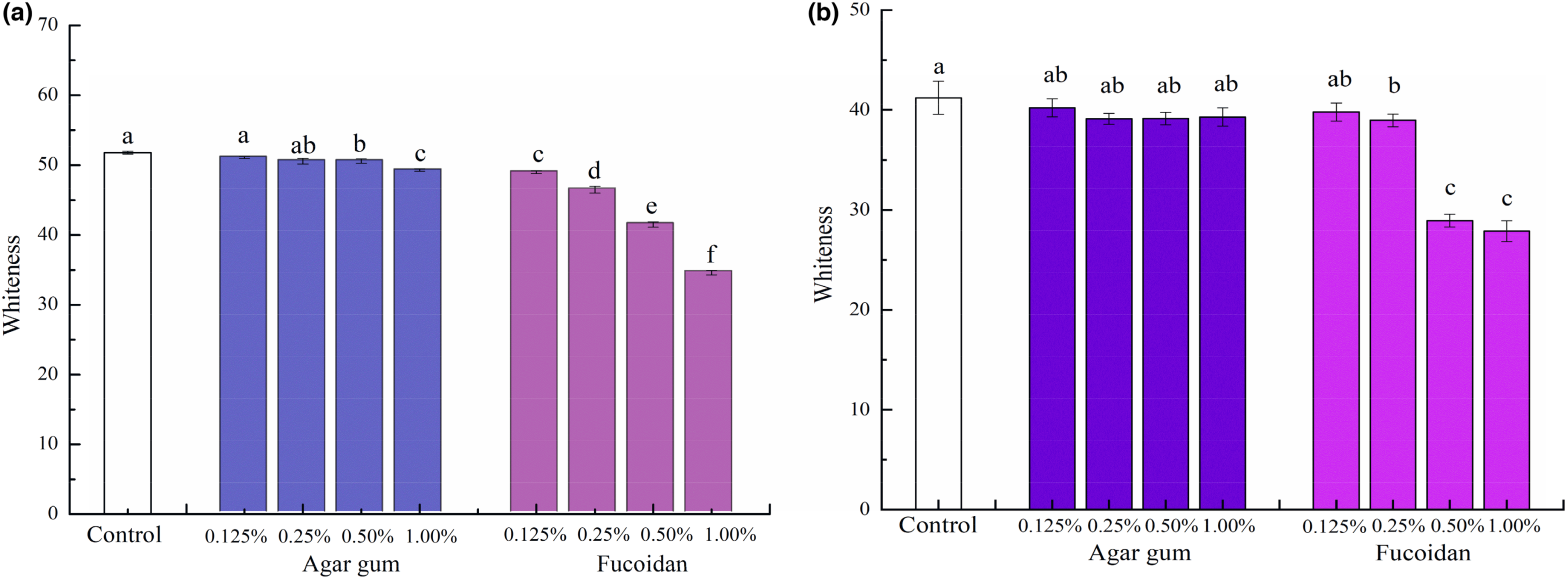
Impacts of agar gum and fucoidan on the whiteness of surimi products without phosphate: (a) at refrigerated storage for 24 h; (b) at frozen storage for 1 year.Control is the surimi products with phosphate. Different lowercase letters in the figure indicate statistical significance between different samples at *p* < .05.

The impacts of agar gum and fucoidan on the whiteness of surimi products are displayed in Figure 3. After refrigerated storage for 24 h (Figure 3a), following the removal of phosphate, the whiteness of surimi products gradually decreased when agar gum was added, which significantly decreased with increasing levels of agar gum up to 0.50% and 1.00% (*p* < .05). Similarly, the addition of fucoidan from 0.125% to 1.00% decreased the whiteness of surimi products (*p* < .05). After frozen storage for 1 year as shown in Figure 3b, the whiteness of surimi products was significantly lower than the refrigerated surimi products (*p* < .05). The agar gum instead of phosphate had no significant effect on the whiteness of surimi products as compared to that with phosphate (*p* ≥ .05), and fucoidan still had a greater impact on the surimi whiteness particularly at >0.25% (*p* < .05). The influence of fucoidan on the whiteness of surimi products was greater than that of agar gum, which may be explained by the presence of brown compounds such as phycophine and xanthophyll in the fucoidan (Zheng et al., 2021).

### 3.5 Impact of agar gum and fucoidan on sensory evaluation of surimi products

Table 2 presents the sensory scores of surimi products with agar gum and fucoidan instead of phosphate. After refrigerated storage for 24 h (Table 2A), agar gum and fucoidan had no significant effect on the odor and taste of surimi products compared with that with phosphate (*p* ≥ .05). Their effects on springiness were consistent with the impacts on the textural properties of the surimi products (Figure 2g–h), and the springiness of the surimi products was maintained when the concentration of agar gum ≤ 0.50% or the concentration of fucoidan ≤ 0.25% (*p* ≥ .05). Agar gum at ≥ 0.50% significantly reduced the color of surimi products (*p* < .05), while fucoidan reduced the color of surimi products in a dose‐dependent manner (*p* < .05), which tend to be in line with the above results of whiteness (Figure 3a). Interestingly, the tissue and fondness of surimi products with 0.125% of fucoidan instead of phosphate increased significantly when compared with the blank group (*p* < .05); the tissues of surimi products with 0.125% agar gum were not significant as compared to the blank group. In contrast, the addition of 1.00% agar gum and fucoidan significantly decreased the score of tissue (*p* < .05). After frozen storage for 1 year (Table 2B), the addition of agar gum and fucoidan had no significant effect on the odor, tissue, springiness, and taste of the surimi products compared with those with phosphate addition (*p* ≥ .05). It may be due to the fact that the gel structure is damaged by the large ice crystals stored for a long time, resulting in the larger pore size of these products, and the difference between them cannot be judged by the naked eye. In addition, agar gum had no effect on the color of surimi products (*p* ≥.05), but the color of surimi products was significantly reduced when fucoidan was added at ≥ 0.50% (*p* < .05). It is worth mentioning that the fondness of surimi products was significantly higher (*p* < .05) when the agar gum concentration was 0.125% than that of the blank group.

**TABLE 2:** Impact of agar gum and fucoidan on sensory evaluation of surimi products without phosphate: (A) at refrigerated storage for 24 h; (B) at frozen storage for 1 year

(A)	Concentration %	Sensory evaluation (score)					
		Color	Odor	Tissue	Springiness	Taste	Fondness
Control	0.00	9.50 ± 0.29^a^	7.87 ± 0.45^a^	7.33 ± 0.62^b^	8.42 ± 0.67^ab^	7.17 ± 0.62^a^	6.58 ± 0.89^b^
Agar gum	0.125	9.25 ± 0.25^a^	8.25 ± 0.75^a^	8.17 ± 0.55^ab^	8.75 ± 0.48^a^	7.50 ± 0.71^a^	8.00 ± 0.65^a^
	0.25	9.17 ± 0.37^a^	8.00 ± 0.41^a^	7.42 ± 0.34^b^	7. 67 ± 0.55^bcd^	7.25 ± 0.95^a^	7.17 ± 0.80^ab^
	0.50	8.67 ± 0.37^b^	8.13 ± 0.45^a^	7.25 ± 0.48^bcd^	7.58 ± 0.61^bcd^	7.58 ± 0.79^a^	7.00 ± 0.65^ab^
	1.00	8.42 ± 0.19^bc^	7.78 ± 0.50^a^	6.25 ± 0.85^de^	7.33 ± 0.75^cde^	7.87 ± 0.71^a^	6.75 ± 0.95^b^
Fucoidan	0.125	8.42 ± 0.34^bc^	8.17 ± 0.37^a^	8.42 ± 0.67^a^	8.33 ± 0.62^abc^	7.58 ± 0.79^a^	8.08 ± 1.06^a^
	0.25	8.00 ± 0.29^cd^	7.83 ± 0.90^a^	7.00 ± 0.65^cde^	7.42 ± 0.79^bcd^	7.78 ± 0.50^a^	7.00 ± 1.00^ab^
	0.50	7.75 ± 0.38^d^	8.17 ± 0.55^a^	6.67 ± 0.75^cde^	6.92 ± 0.98^de^	7.27 ± 0.49^a^	6.75 ± 0.85^b^
	1.00	7.33 ± 0.37^e^	8.30 ± 0.41^a^	6.08 ± 1.24^e^	6.33 ± 1.11^e^	7.67 ± 0.55^a^	6.50 ± 0.82^b^
(B)							
Control	0.00	9.25 ± 0.69^a^	5.67 ± 0.94^a^	6.25 ± 1.15^a^	8.17 ± 0.90^a^	7.45 ± 1.23^a^	6.08 ± 1.30^b^
Agar gum	0.125	8.92 ± 0.45^a^	6.83 ± 1.70^a^	7.33 ± 0.90^a^	8.50 ± 0.82^a^	8.03 ± 0.87^a^	7.73 ± 0.80^a^
	0.25	8.33 ± 0.80^abc^	5.87 ± 1.07^a^	6.92 ± 0.98^a^	7.80 ± 1.48^a^	8.12 ± 0.73^a^	7.05 ± 1.20^ab^
	0.50	8.42 ± 1.10^ab^	6.42 ± 1.37^a^	6.08 ± 1.48^a^	7.25 ± 1.46^a^	7.58 ± 1.30^a^	7.08 ± 1.30^ab^
	1.00	8.33 ± 1.14^abc^	6.50 ± 1.50^a^	7.03 ± 1.00^a^	7.33 ± 1.52^a^	7.80 ± 1.03^a^	6.55 ± 1.04^ab^
Fucoidan	0.125	8.75 ± 0.99^ab^	6.00 ± 1.32^a^	6.80 ± 1.24^a^	8.45 ± 1.19^a^	7.50 ± 1.08^a^	7.17 ± 0.85^ab^
	0.25	8.50 ± 0.96^ab^	6.42 ± 1.48^a^	6.67 ± 1.07^a^	8.00 ± 0.82^a^	8.08 ± 0.73^a^	6.58 ± 0.84^ab^
	0.50	7.50 ± 0.91^bc^	5.75 ± 1.46^a^	6.50 ± 1.12^a^	8.33 ± 0.75^a^	7.92 ± 0.93^a^	6.92 ± 0.84^ab^
	1.00	7.08 ± 1.02^c^	6.33 ± 1.07^a^	6.25 ± 1.41^a^	6.92 ± 1.30^a^	8.00 ± 1.29^a^	6.95 ± 1.33^ab^

*Note*: Control is the surimi products with phosphate. ^†^Different superscript lowercase letters in the same column indicate statistical significance between different samples at *p* < .05.

Overall, agar gum and fucoidan at 0.125% instead of phosphate can maintain the gel properties and sensory quality of surimi products. Such effects of agar gum and fucoidan were almost similar between surimi products in refrigerated storage for 24 h and in frozen storage for 1 year. Therefore, the following structural changes were determined only for surimi products with refrigerated storage for 24 h.

### 3.6 Impacts of agar gum and fucoidan on the protein structure of surimi products

**FIGURE 4:**
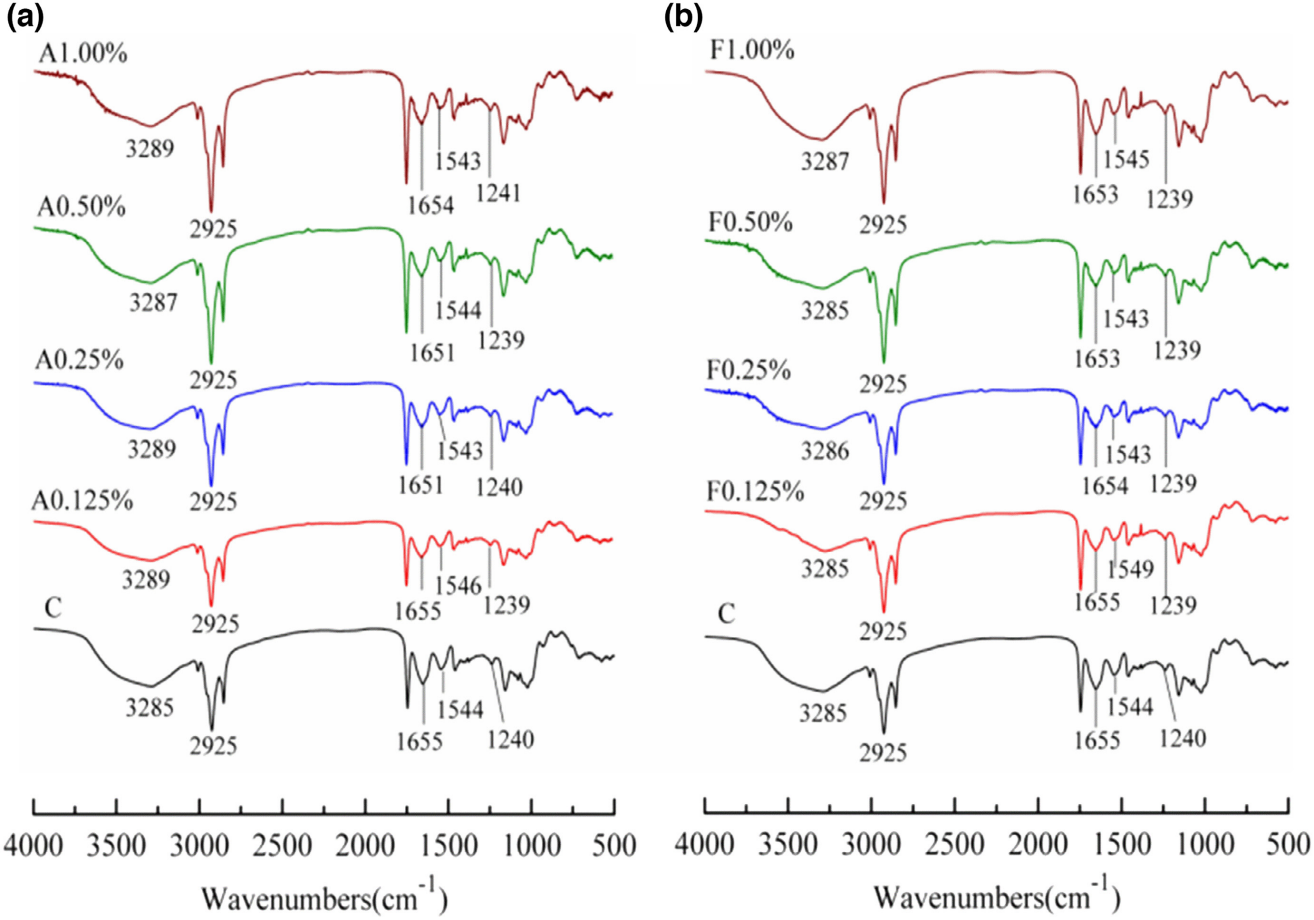
Impacts of agar gum and fucoidan on FT‐IR spectra of surimi products without phosphateC represents the control surimi products with phosphate; a 0.125%, a 0.25%, a 0.50%, and a 1.00% represent the surimi products with 0.125%, 0.25%, 0.50%, and 1.00% of agar gum, respectively; F 0.125%, F 0.25%, F 0.50%, and F1.00% represent the surimi products with 0.125%, 0.25%, 0.50%, and 1.00% of fucoidan, respectively.

FT‐IR spectroscopy is an effective method for evaluating relative changes in protein secondary structure (Zhao et al., 2020). The impacts of agar gum and fucoidan on protein secondary structure of surimi products without phosphate were analyzed by the FT‐IR spectra and the results are shown in Figure 4 and Table 3. The control surimi products with phosphate exhibited characteristic peaks at 3285 and 2925 cm^−1^, corresponding to amide A and amide B arising from N‐H and C‐N stretching vibrations, respectively (Fang et al., 2021). The absorption peaks of amides I, II, and III in surimi products with phosphate were found at 1655, 1544, and 1240 cm^−1^, respectively, according to the previous study (Guan et al., 2018). With the addition of agar gum and fucoidan to replace phosphate, the above characteristic peaks were observed and no new peaks were found in the FT‐IR spectra of surimi products, indicating that the rigid protein structure formed in surimi products with agar gum and fucoidan was similar to that in surimi products with phosphate. Furthermore, the variation in the ratio of protein secondary structure motifs including *α*‐helix, *β*‐sheet, *β*‐turn, and random coils was tested by deconvolution and Gaussian fitting in the related wavenumber range of 1650–1660, 1600–1640, 1660–1700, and 1640–1650 cm^−1^ (Barth, 2007). As shown in Table 3, the results demonstrated that the ratios of *α*‐helix, *β*‐sheet, and *β*‐turn structure motifs of the surimi products with agar gum and fucoidan added were the same as the surimi products with phosphate (*p* ≥ .05). Our results suggested that using agar gum and fucoidan to replace phosphate did not affect the protein structure of surimi products.

**TABLE 3:** The secondary protein structure percentage of surimi products based on FT‐IR analysis

	α‐Helix(%)	β‐Sheet(%)	β‐Turn(%)	Random coils(%)
C	21.85 ± 0.44^a^	29.47 ± 0.82^a^	28.48 ± 0.43^a^	20.20 ± 0.08^abc^
A 0.125%	21.91 ± 0.32^a^	29.00 ± 0.54^a^	28.88 ± 0.48^a^	20.22 ± 0.00^abc^
A 0.25%	21.93 ± 0.22^a^	29.46 ± 0.62^a^	28.51 ± 0.63^a^	20.10 ± 0.17^bc^
A 0.50%	21.82 ± 0.13^a^	28.95 ± 0.52^a^	28.75 ± 0.40^a^	20.49 ± 0.18^a^
A 1.00%	21.70 ± 0.09^a^	28.81 ± 0.36^a^	29.08 ± 0.47^a^	20.40 ± 0.16^ab^
F 0.125%	21.43 ± 0.18^a^	29.61 ± 0.21^a^	29.27 ± 0.39^a^	19.69 ± 0.25^c^
F 0.25%	21.54 ± 0.20^a^	28.71 ± 0.45^a^	29.30 ± 0.35^a^	20.45 ± 0.09^ab^
F 0.50%	21.53 ± 0.45^a^	29.74 ± 1.03^a^	28.44 ± 0.57^a^	20.29 ± 0.12^ab^
F 1.00%	21.37 ± 0.16^a^	29.88 ± 0.69^a^	28.82 ± 0.83^a^	19.93 ± 0.24^c^

*Note*: Different superscript lowercase letters (a, b, c) in the same column indicate statistical significance between different samples at p < .05. C represents the control surimi products with phosphate; A 0.125%, A 0.25%, A 0.50%, and A 1.00% represent the surimi products with 0.125%, 0.25%, 0.50%, and 1.00% of agar gum, respectively; F 0.125%, F 0.25%, F 0.50%, and F1.00% represent the surimi products with 0.125%, 0.25%, 0.50%, and 1.00% of fucoidan, respectively.

### 3.7 Impacts of agar gum and fucoidan on protein compositions and patterns of surimi products

**FIGURE 5:**
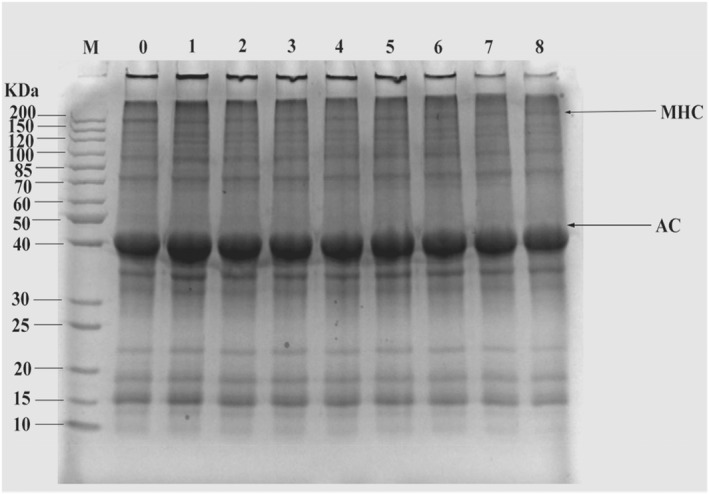
Impacts of agar gum and fucoidan on SDS‐PAGE of surimi products without phosphateM: Standard protein marker; 0: Surimi with phosphate; 1, 2, 3, and 4: Surimi with 0.125%, 0.25%, 0.50%, and 1.00% of agar gum, respectively; 5, 6, 7, and 8: Surimi with 0.125%, 0.25%, 0.50%, and 1.00% of fucoidan, respectively; MHC: Myosin heavy chain; AC: Actin.

Protein compositions and patterns of surimi products were analyzed via SDS‐PAGE and the results are shown in Figure 5. The typical bands of myosin heavy chain (MHC, around 200 kDa) and actin (AC, around 43 kDa) were visible for all samples following SDS‐PAGE (Zhuang et al., 2017). The addition of agar gum and fucoidan had no significant effect on the intensities of AC bands, indicating that AC maybe not involved in the myosin cross‐linking as suggested previously (Huang et al., 2021). However, the intensity of MHC could be changed by the addition of agar gum and fucoidan. The MHC bands of surimi products with 0.125% of agar gum and fucoidan were darker than the control with phosphate. This result suggested that the addition of 0.125% of agar gum and fucoidan reinforced the surimi gel network more effectively than phosphate. As compared to surimi products with 0.125% of agar gum and fucoidan, the band intensity of the MHC became weaker when the added concentration of agar gum and fucoidan was up to ≥ 0.50%. In particular, the aggregates on the top of the concentrated gel were greatly decreased during SDS‐PAGE of surimi products with fucoidan ≥ 0.50% added, suggesting that seaweed polysaccharides, especially fucoidan with a high concentration ≥ 0.50%, could inhibit the cross‐linking and aggregation of proteins (Zhou et al., 2021).

### 3.8 Impacts of agar gum and fucoidan on the microstructure of surimi products

**FIGURE 6:**
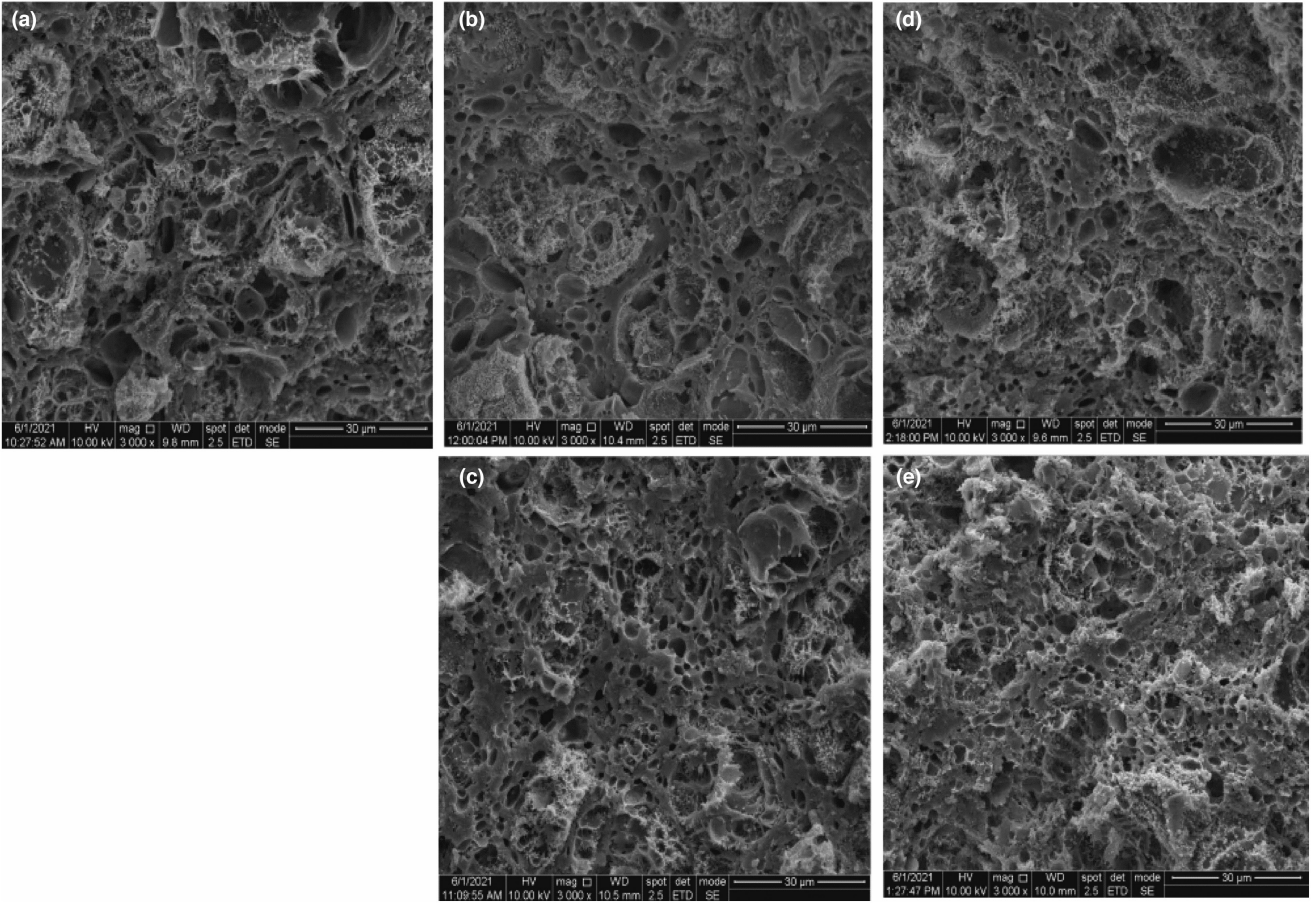
Impacts of agar gum and fucoidan on ESEM images of surimi products without phosphate: (a) control surimi with phosphate; (b) surimi with 0.125% of agar gum; (c) surimi with 0.125% of fucoidan; (d) surimi with 1.00% of agar gum; and (e) surimi with 1.00% of fucoidan.

The impacts of 0.125% and 1.00% of agar gum and fucoidan on the microstructure of surimi products without phosphate are displayed in Figure 6. The control surimi gel with phosphate (Figure 6a) had a porous gel network, whereas the pore sizes of the surimi product gel network were smaller and more uniform resulting in a more compact three‐dimensional structure following the addition of 0.125% agar gum (Figure 6b) and 0.125% fucoidan (Figure 6c) to replace phosphate. Sodium pyrophosphate among phosphate compounds has been reported to form an ordered structure with finer strands (Julavittayanukul et al., 2006; Lee et al., 2018). In our results, 0.125% agar gum and fucoidan could replace phosphate to strengthen the network structure of surimi products. This phenomenon may be due to the fact that a low concentration of seaweed polysaccharides can promote the cross‐linking of proteins (Zheng et al., 2021). The results are consistent with the findings of SDS‐PAGE. Moreover, Zhao et al. (2019) also reported that an appropriate amount of regenerated cellulose fiber promoted the formation of a tighter and uniform three‐dimensional network structure of the myofibril protein gel. Compared with the gel network of the surimi products with 0.125% of agar gum, the addition of 0.125% of fucoidan to the surimi products showed a larger pore size and weaker ordered network structure, which was also highly consistent across the findings of the above‐mentioned SDS‐PAGE analysis.

In addition, the pores of the surimi products with 1.00% agar gum (Figure 6d) or 1.00% fucoidan (Figure 6e) were more uneven, and the gel network structure was much looser as compared with the control and the surimi products with 0.125% of agar gum (or fucoidan) added to replace phosphate (Figure 6a–c). It is reasonable to speculate that the high concentration of seaweed polysaccharides could hinder the cross‐linking between proteins, which was also confirmed by the decreased gel strength and the change in protein patterns using SDS‐PAGE analysis. A similar phenomenon was also observed in a previous report where an excess addition of psyllium husk caused the destruction of the gel network structure of myofibrillar protein (Zhou et al., 2021).

## 4 CONCLUSIONS

Agar gum and fucoidan at an appropriate concentration instead of phosphate maintained the gel properties of surimi products. Following the addition of 0.125% agar gum and fucoidan, the WHC, gel strength, and textural properties of surimi products were equal to the control. Especially at frozen storage for 1 year, 0.125% of agar gum reduced expressible moisture content of surimi products by around 10% (*p* < .05). Moreover, enhanced cross‐linking and aggregation of proteins, as well as a denser gel network structure were found in these surimi products based on the results of SDS‐PAGE analysis and ESEM observations. At high concentrations, the addition of agar gum and fucoidan (> 0.50%) greatly increased the WHC but decreased the gel strength and textural properties, for example, the springiness of surimi products (*p* < .05). Particularly, 1.00% agar gum and fucoidan decreased the gel strength of surimi products by around 20% (*p* < .05). SDS‐PAGE and ESEM analyses showed that cross‐linking and aggregation of surimi proteins were inhibited by excess addition of seaweed polysaccharides and that the gel network structure of surimi proteins was looser. This may be due to the negative effect of agar gum and fucoidan on surimi products at higher concentrations. Our findings suggest the potential application of 0.125% agar gum and fucoidan in surimi products to replace phosphate. The results can broaden the application of these seaweed polysaccharides and provide new strategies for developing surimi products without phosphate.

## CONFLICT OF INTEREST

The authors declare no conflict of interest.

## Data Availability

Research data are not shared.
